# Combinatorial Treatment of DNA and Chromatin-Modifying Drugs Cause Cell Death in Human and Canine Osteosarcoma Cell Lines

**DOI:** 10.1371/journal.pone.0043720

**Published:** 2012-09-05

**Authors:** Venugopal Thayanithy, ChangWon Park, Aaron L. Sarver, Reena V. Kartha, Derek M. Korpela, Ashley J. Graef, Clifford J. Steer, Jaime F. Modiano, Subbaya Subramanian

**Affiliations:** 1 Department of Surgery, University of Minnesota, Minneapolis, Minnesota, United States of America; 2 Department of Medicine, University of Minnesota, Minneapolis, Minnesota, United States of America; 3 Masonic Cancer Center, University of Minnesota, Minneapolis, Minnesota, United States of America; 4 Department of Experimental and Clinical Pharmacology, College of Pharmacy, University of Minnesota, Minneapolis, Minnesota, United States of America; 5 Department of Veterinary Clinical Sciences, University of Minnesota, St. Paul, Minnesota, United States of America; 6 Department of Genetics, Cell Biology & Development, University of Minnesota, Minneapolis, Minnesota, United States of America; Institute of Clinical Physiology, c/o Toscana Life Sciences Foundation, Italy

## Abstract

Downregulation of microRNAs (miRNAs) at the 14q32 locus stabilizes the expression of cMYC, thus significantly contributing to osteosarcoma (OS) pathobiology. Here, we show that downregulation of 14q32 miRNAs is epigenetically regulated. The predicted promoter regions of miRNA clusters at 14q32 locus showed no recurrent patterns of differential methylation, but Saos2 cells showed elevated histone deacetylase (HDAC) activity. Treatment with 4-phenylbutyrate increased acetylation of histones associated with 14q32 miRNAs, but interestingly, robust restoration of 14q32 miRNA expression, attenuation of cMYC expression, and induction of apoptosis required concomitant treatment with 5-Azacytidine, an inhibitor of DNA methylation. These events were associated with genome-wide gene expression changes including induction of pro-apoptotic genes and downregulation of cell cycle genes. Comparable effects were achieved in human and canine OS cells using the HDAC inhibitor suberoylanilide hydroxamic acid (SAHA/Vorinostat) and the DNA methylation inhibitor Zebularine (Zeb), with significantly more pronounced cytotoxicity in cells whose molecular phenotypes were indicative of aggressive biological behavior. These results suggested that the combination of these chromatin-modifying drugs may be a useful adjuvant in the treatment of rapidly progressive OS.

## Introduction

Osteosarcoma (OS), the most common primary tumor of bone, principally affects children and adolescents. Progression of disease is inexorable and response to therapy can be unrewarding: fewer than 50% of patients live beyond 10 years, and there are no reliable predictors to guide the choice or intensity of therapy [1], [2]. OS is cytogenetically chaotic [3] and recurrent chromosomal aberrations [4]–[6] have been found that appear to be etiologically significant, but they do not define OS subtypes with distinct biological behavior. We recently showed that multiple microRNAs (miRNAs) present at the 14q32 locus are downregulated in human OS compared to normal bone tissue [7], [8].

Expression of miRNAs can be altered by changes in transcription, by epigenetic events, and by DNA copy number aberrations, creating imbalances in miRNA-gene regulatory networks [9], [10]. Chromosome 14q32 locus represents one of the most miRNA-enriched regions in the human genome, containing >40 distinct miRNAs. These miRNAs are grouped into at least four clusters; and the locus appears to be involved in several diseases [11], including lymphoblastic leukemias [12], high-grade ovarian tumors [13], gastrointestinal stromal tumors [14], and colon cancer [15]. Homologous regions of these miRNA clusters in mouse are highly conserved [16] and imprinted maternally and paternally. Deletion of the non-coding RNA, Gtl2, with its differentially methylated region induces lethal parent-origin-dependent developmental defects [17]. The 14q32 miRNA locus is not recurrently deleted in human OS [7] but its regulation remains incompletely understood.

In addition to demonstrating downregulation of 14q32 miRNAs in human OS, we have also described differentially expressed gene signatures that stratify this cancer into two groups with distinct biological behavior [18], [19]. Both events appear to be temporally related; that is, downregulation of 14q32 miRNAs is inversely associated with expression of genes that regulate cell cycle progression and are directly correlated with expression of pro-apoptotic genes. However, it is not yet established if these events are functionally linked, and their mechanistic basis is unclear. Here, we examined the role of epigenetic events to control transcription of 14q32 miRNAs in OS, and we tested the hypothesis that restoring their expression using DNA and chromatin-modifying drugs would re-establish physiologic gene expression and promote OS cell apoptosis.

## Materials and Methods

### Cells

Human Saos2 (ATCC #HTB-85) and U2OS (ATCC #HTB-96) cells were a gift from Dr. Richard Gorlick, Albert Einstein College of Medicine, New York, USA. Procedures for establishing growth characteristics of the OSCA-32, OSCA-40 and OSCA-78 canine OS cell lines were described earlier [20], [21]. Human Saos2 cells were grown in McCoy's 5A Medium with 15% fetal bovine serum at 37°C in a humidified atmosphere with 5% CO_2_. For cytotoxicity assays, human Saos2 and U2OS cells, and canine OSCA-32, OSCA-40 and OSCA-78 cells were cultured in Dulbecco's minimal essential medium supplemented with 10% fetal bovine serum at 37°C in a humidified atmosphere with 5% CO_2_.

### Chemicals and reagents

Chemicals and reagents were purchased from Sigma Aldrich (St. Louis, MO) unless otherwise specified.

### DNA and RNA extractions

Frozen OS tissue samples were obtained from Bionet, the tissue biorepository of the University of Minnesota. The University of Minnesota, Institutional Review Board committee headed by Dr. Timothy Mulcahy approved this study based on exempt-4 classification (approval number #0801E25485). The IRB determined that the referenced study is exempt from review under federal guidelines 45 CFR Part 46.101(b) category #4 existing data; records review; pathological specimens. Genomic DNA was isolated from patient samples using Gentrapure DNA isolation kit (Qiagen, Valencia, CA) following the manufacturer's protocol. Total RNA was isolated from Saos2 cells using the mirVana total RNA isolation kit (Ambion Inc, Austin TX). RNA was quantified using Nanodrop 8000 (Nanodrop Technologies LLC, Wilmington, DE) spectrophotometer. Quality of DNA and RNA were assessed by agarose gel electrophoresis analysis. A total of nine OS patient tissue samples (FT-3, FT-4, FT-7, FT-8, FT-10, FT-11, FT-12, FT-17, FT-19) and 3 normal bone tissue samples (FT-401, FT-402, FT-403) were used. Clinical information about these patient samples is reported in our earlier study [7].

### DNA methylation

Bisulfite treatment of genomic DNA was performed as described [22]. Briefly, genomic DNA was digested with *Hind* III and purified by phenol-chloroform extraction followed by ethanol precipitation. Four micrograms of digested DNA were incubated in bisulfite solution (4.4 M sodium bisulfite and 10 mM hydroquinone) at 55°C in the dark for 15 hrs. DNA was then purified using the Wizard® DNA clean-up system (Promega, Madison, WI), incubated in 0.3 M NaOH for 20 min at 37°C for desulfonation, and finally precipitated by ethanol. Bisulfite-treated genomic DNA was dissolved in 30 µl of distilled water, and 2 µl of this DNA were amplified in 50-µl PCR reactions. Bisulfite-mediated PCR primers (Table S1) were designed according to the DNA sequences converted *in silico* by Methprimer [23]. The PCR reaction consisted of initial denaturation at 94°C for 7 min followed by 35 cycles of 20 sec at 94°C, 20 sec at 54°C∼56°C, and 45 sec at 72°C, with a final extension of 5 min at 72°C. PCR components were based on the Expand High Fidelity PCR system (Roche, Branchburg, NJ) and were initiated with a hot start.

### Combined bisulfite restriction analyses (COBRA)

COBRA [24] was performed with the PCR products from bisulfite-treated DNA templates. Restriction enzymes whose recognition sites contained at least one CpG dinucleotide included: *Hpy*CH4I V (5′-ACGT-3′); *Bst*UI (5′-CGCG-3′); and *Taq*I (5′-TCGA-3′). Restriction endonuclease(s) were chosen for each region, depending on the number of possible recognition sites in the Methprimer-converted sequence of the region using miRNA specific oligos (Table S1). *Alu*I (5′-AGCT-3′) and *Mse*I (5′-TTAA-3′) were used to verify the completeness of bisulfite-mediated conversion of unmethylated cytosines. *Alu*I sites in the original sequence would be eliminated by bisulfite treatment and new *Mse*I sites that were absent from the original sequence would be created by this treatment. Methylated regions would then generate new restriction sites for *Bst*UI or *Hpy*CH4IV. Digested products along with undigested controls were separated by 1.2% agarose gel electrophoresis. The pGEMT vector (GE Healthcare, Piscataway, NJ) was used as control DNA to monitor the activity of restriction enzymes (Figure S1).

### Western blotting

For acetylated-Histone-3 immunoblotting, cells were trypsinized, washed twice in PBS and lysed in M-PER protein extraction reagent (Thermo Scientific, Rockford, IL) as recommended by the manufacturer. For c-MYC immunoblotting, cells were harvested and lysed in a buffer containing 25 mM HEPES, pH 7.6, 300 mM NaCl, 1.5 mM Mgcl2, 0.2 mM EDTA and 0.1% Triton X-100 with protease inhibitor cocktail. Total protein was estimated by the Bradford method (BioRad, Hercules, CA). Total cellular protein (15–20 µg) in Laemmli buffer was denatured at 65°C for 5 min and resolved on precast 10% Tris-HCl Criterion gels (Mini-Protean TGX, BioRad). Proteins were transferred to PVDF membranes for blotting. Membranes were incubated with a polyclonal anti-Ac-Histone H3 antibody (Millipore, Danvers, MA) raised against a peptide corresponding to the first 20 amino acids of histone-H3 containing K4, K9 and K14 (where K9 and K14 were acetylated), anti-cMYC mouse monoclonal antibody (Santa Cruz Biotechnology, Santa Cruz, CA), or anti-GAPDH antibodies (Santa Cruz or Abcam, Cambridge, MA). Detection was done using chemiluminescence (GE Healthcare) or with the Odyssey Li-COR imager (Lincoln, NE).

### Chromatin immunoprecipitation (ChIP)

ChIP assays were performed using the Chromatin immunoprecipitation assay kit (Millipore) following the manufacturer's instructions. Briefly, cells (1.0×10^6^) were cross-linked with 1% formaldehyde at 37°C for 10 min, washed twice in cold PBS with protease inhibitors, lysed in SDS buffer, sonicated, and cleared by centrifugation. The supernatants were diluted in ChIP dilution buffer, pre-cleared in protein-G sepharose, and immunoprecipitated with anti-Ac-Histone H3 antibody bound to protein-G sepharose. Immune complexes were disrupted by incrementally increasing the salt concentrations and eluted in TE buffer. Prior to PCR reactions, cross linking of DNA was reversed, proteins were digested using Proteinase K, and DNA was purified using routine phenol/chloroform organic extraction. Eluted DNA was amplified with miRNA specific oligonucleotides corresponding to a stretch of 200 nucleotide between positions +1 to −200 (Table S2) using QuantiTect SYBR Green PCR Kit (Qiagen) in an ABI 7500 optical cycler (Applied Biosystems, Foster City, CA). Lysates where the antibody was omitted were used as controls for background subtraction. Oligonucleotides spanning 166 nucleotides on the GAPDH promoter and binding to the intergenic region between the GAPDH gene and the chromosome condensation-related SMC-associated protein (CNAP1) were used as positive and negative controls, respectively (Active Motif, Carlsbad, CA).

### Treating cells with inhibitors of DNA methylation and histone deacetylation

5-Aza-2′-deoxycytidine (5-AzadC, #A3656, Sigma Aldrich) was dissolved in water (0.25 mg/ml) immediately before use, and 4-Phenyl-N-Butyric acid, (4-PBA, #P21005, Sigma Aldrich) was dissolved in 50% ethanol as a 20 mg/ml solution and stored at −20°C. Saos2 cells (0.7×10^6^) seeded in T75 flasks were treated with a combination of drugs with or without Z-VAD-fmk (15 µM). Cells were grown under standard conditions; fresh medium containing the drug was replaced every 12 hrs; and the cells were treated continuously for 6 days as reported earlier [25]. Similarly, Saos2, U2OS as well as canine OSCA-40, OSCA-78, and OSCA-32 cells were cultured in the presence of Suberoylanilide Hydroxamic Acid or N-hydroxy-N′-phenyl-octanediamide (SAHA) (1, 5 or 10 µM) and Zebularine or 1-(β-D-Ribofuranosyl)-1,2-dihydropyrimidin-2-one, 2-Pyrimidone-1-β-D-riboside (Zeb) (1, 10 or 100 µM) separately or in combination as indicated and cell viability was measured 48 hrs after treatment. Each experiment was repeated at least 3 times and representative results are shown.

### qRT-PCR

Total RNA was subjected to polyadenylation and reverse transcription using the miRscript reverse transcription kit (Qiagen). cDNA was quantified with the miRscript SYBR green detection kit (Qiagen) using a miRNA-specific forward primer and a universal primer, following the manufacturer's instructions, in an ABI 7500 optical cycler (Applied Biosystems). Oligonucleotide primer sequences used for analysis are provided in Table S3. *U6 snRNA* was used as controls for miRNA qRT-PCR analyses. Threshold cycle (Ct) values calculated by the SDS v1.2.1 software (Applied Biosystems) were used for statistical analysis. Expression was calculated following the delta-delta Ct (2^−ΔΔ*C*^_T_) method [26].

### Cell viability

Cell viability was tested by exclusion of vital dyes using the Live/Dead, viability/cytotoxicity assay (Molecular Probes, Eugene, OR) and the MTS assay (Promega, Madison, WI) 48 hr after treatment. Cytotoxicity was measured using the LDH cytotoxicity detection kit (Clontech, Mountain View, CA) 10 hr after treatment.

### Apoptosis assay

The rate of apoptosis was determined by flow cytometry following the method of Riccardi and Nicoletti [27]. Briefly cells were stained with a sub saturation quantity of propidium iodide (PI) and data were collected (n = 10,000) as recommended by flow cytometry 48 hr after treatment in a FACS Calibur (BD Biosciences, San Jose, CA).

### ZVAD-fmk treatment

For apoptosis inhibition experiments, cells were continuously incubated with 20 µM of ZVAD-fmk (BD Biosciences) immediately after drug treatment and until analysis.

### mRNA expression profiling

Gene expression profiles were assessed using the human HT-12 Beadchip platform (Illumina Inc., San Diego, CA) [28]. Data were analyzed using GeneData Expressionist Software (GeneData Inc, San Francisco, CA); genes that showed statistically significant differences in expression between groups were determined using the two-group *t*-test and ANOVA.

## Results

### Osteosarcoma samples do not show consistent changes in methylation patterns in the 14q32 miRNA locus

**Figure 1:**
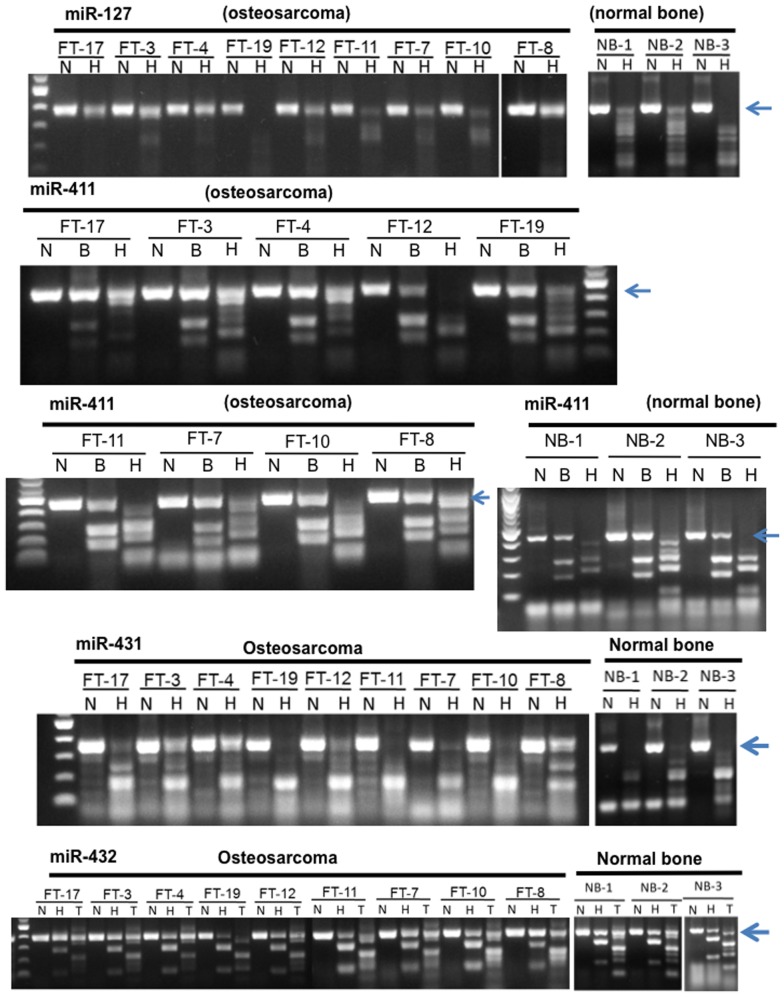
COmbined Bisuifite Restriction Analysis (COBRA) for CpG regions upstream of miR-127, -411, -431 and -432 in OS and normal bone tissue samples. The following enzymes were used as indicated (B-*Bst* UI; H-*Hpy* CH4IV; T-*Taq* I; N- no enzyme). Bisulfite conversion of methylated CpGs created new restriction sites and the level of digestion of the bisulfite converted PCR product with listed enzymes was directly related to its intrinsic methylation, hence indicative of level of DNA methylation. As a reference for normal DNA methylation status, identical analysis for each region was performed with DNA from normal bone tissues. Relative difference in the digestion insensitivity of the PCR product (marked by arrow) in OS patients relative to normal bone tissue was indicative of similar or relative hypomethylation of these loci in OS patients.

Our previous work showed that a cluster of miRNAs in the 14q32 chromosomal locus was significantly downregulated in human OS tumor tissues and cell lines compared to normal bone tissues and cell lines [7], [8]. We further showed that downregulation of 14q32 miRNAs was not due to recurrent deletion of this locus [7]; thus, we investigated the possibility that downregulation of 14q32 miRNAs was controlled epigenetically through DNA methylation and histone acetylation. We tested DNA methylation at the CpG regions upstream of selected 14q32 miRNA transcripts (miR-127, -411, -431 and -432) [11]. Bisulfite mediated conversion creates new sites for *Taq*I (T) or *HpyCH4* IV and methylated CpG sites will be digested by these enzymes after bisulfite conversion; and thus, the levels of digestion are a direct measure of positive intrinsic methylation [24]. *Taq*I (T), *HpyCH4* IV (H), and *BstU*I (B) restriction enzymes were used to digest the bisulfite converted DNA and pGEMT plasmid vector was used as control DNA to monitor the bisulfite conversion and restriction digestion (Figure S1). The presence of bisulfite converted PCR products that were insensitive to digestion suggested no change or relative hypomethylation in upstream regions of miR-127 and -411 in OS patients and corresponding age matched normal bone tissues (Figure 1). Similarly, methylation patterns of the individual OS samples showed either no significant change or partial hypomethylation patterns at miR-431, miR-432 upstream regions, relative to normal bone tissues (Figure 1). Taken together DNA methylation analyses of upstream sequences of selected 14q32 miRNAs suggested that OS patient samples were characterized predominantly by hypomethylation in CpG sites upstream of these miRNAs. Thus DNA methylation pattern observed at these 14q32 miRNA regions did not support the differential expression of these miRNAs in normal bone and OS tumor tissues, suggesting that DNA methylation may not be a primary factor attributing to the downregulation of these miRNAs in human OS.

### Downregulation of 14q32 miRNAs is associated with histone deacetylation in OS cells

**Figure 2:**
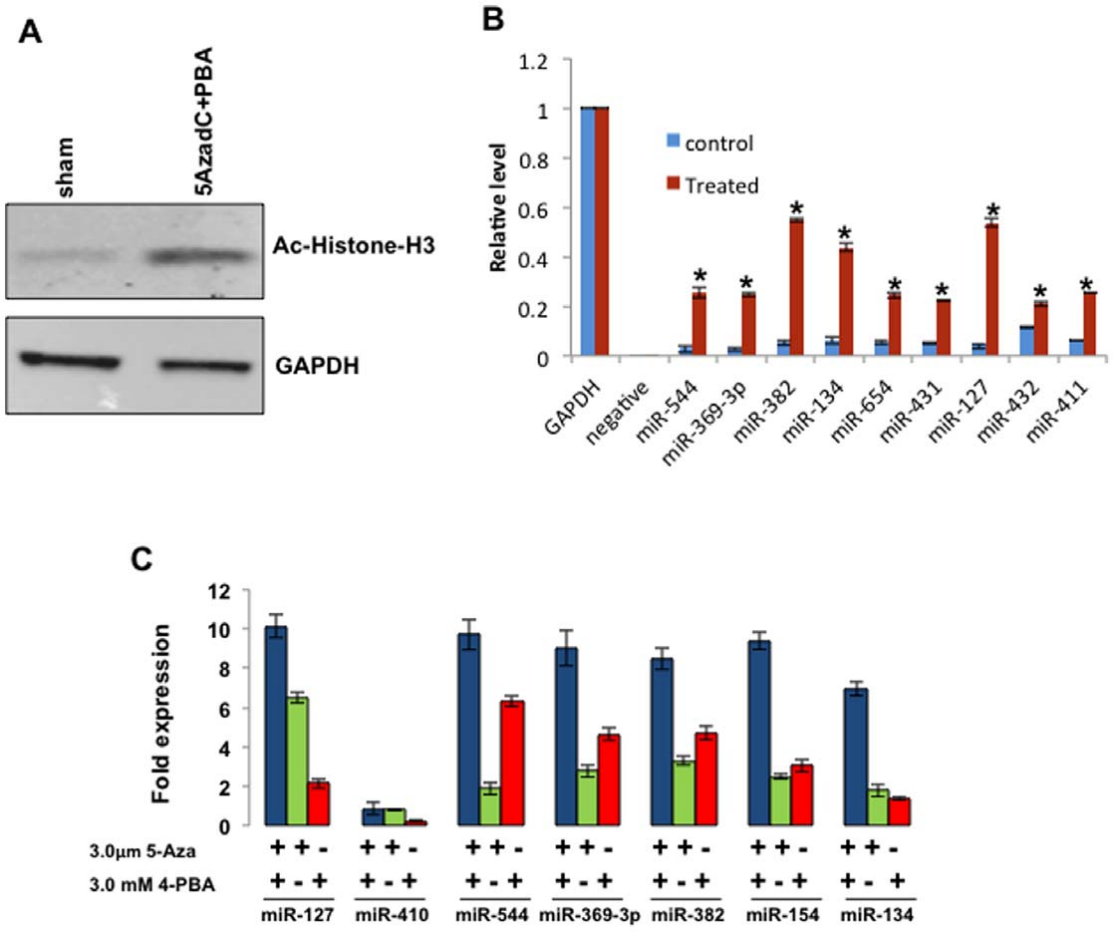
5-AzadC and 4-PBA activates 14q32 miRNAs expression and destabilize cMYC in Saos2 cells. **A**) 5-AzadC and 4-PBA treated Saos2 cells were subjected to western blot analysis with Anti-acetylated histone-H3 antibody; and GAPDH is shown as control. Note an increase in the level of acetylated Histone-H3 in the treated cells. **B**) 5-AzadC and 4-PBA significantly increased the acetylation of histone-H3 of representative 14q32 miRNA upstream regions (−200 bp); *p values <0.005. **C**) Quantitative RT-PCR analysis of 14q32 miRNAs following the treatments with 3 µM 5-AzadC and 3 mM 4-PBA. Saos2 cells were treated with 3.0 µM 5-AzadC and 3.0 mM 4-PBA for 6 days, unless otherwise mentioned.

We next examined histone acetylation using Saos2 cells as an OS model. Immunoblotting showed histone H3 proteins were largely deacetylated in these cells (Figure 2A). Previous experiments in bladder cancer cells showed that a combination of epigenetic modifiers, including inhibitors of histone deacetylation such as sodium-4-phenylbutyrate (4-PBA) and inhibitors of DNA methylation such as 5-Azacytidine (5-AzadC), restored expression of miR-127 at the 14q32 locus [25]. Hence, we examined if these drugs could similarly reverse histone deacetylation and restore 14q32 miRNA expression in Saos2 cells. Figure 2A shows that global acetylation of histone-H3 increased following treatment of cells with 5-AzadC (3 µM) and 4-PBA (3 mM). We used ChIP and qPCR to assess association of acetylated histone H3 proteins with upstream genomic sequences of 14q32 miRNAs. Immune complexes were precipitated with anti-acetylated-Histone 3 antibody from cells treated with vehicle or from cells treated with the combination of 5-AzadC and 4-PBA. Sequences 200 nucleotides upstream to the transcription start site of miR-654, miR-431, miR-127, miR-432, miR-411, miR-544, miR-369-3p, miR-382 and miR-134 were then amplified using qPCR from DNA present in the immune complexes. Figure 2B shows that the fraction of acetylated histone H3 bound to the upstream sequences of 14q32 miRNAs was significantly increased (p<0.005) in the treated cells.

Next, we determined if histone H3 re-acetylation led to increased expression of 14q32 miRNAs. Saos2 cells were treated with vehicle or with the combination of 5-AzadC and 4-PBA as described above, and expression of representative 14q32 miRNAs was analyzed using qRT-PCR. Figure 2C shows that for six of seven miRNAs tested, expression was significantly increased in the treated cells. Neither drug alone was able to consistently increase expression of these miRNAs, nor did the observed increases reach the same magnitude as those seen when the two drugs were used in combination.

### Restoring expression of 14q32 miRNAs in Saos2 cells was functionally significant

**Figure 3:**
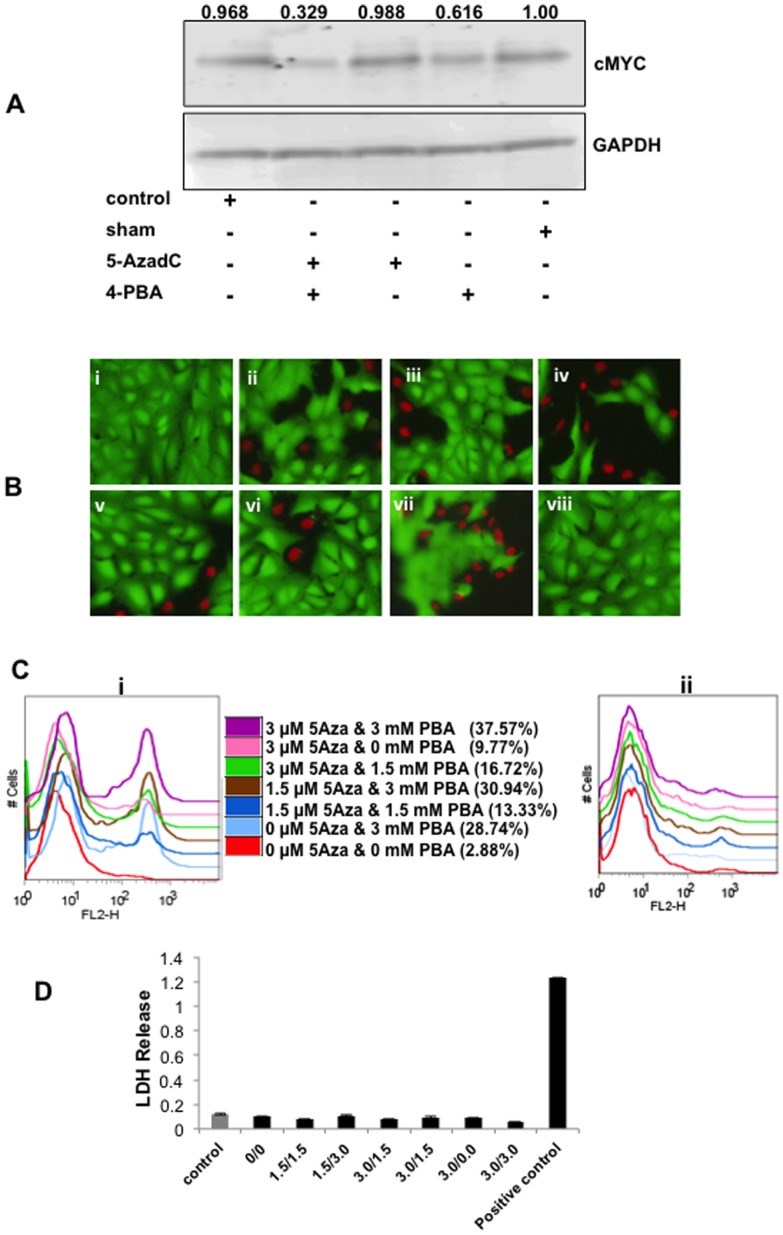
5-AzadC and 4-PBA induce apoptosis in Saos2 cells. **A**) Cells treated with 5-AzadC and 4-PBA alone or in combination along with sham and untreated cells were subjected to western blot analysis using anti-cMYC antibody. The blot was stripped and re-probed with anti-GAPDH antibody. Signal intensity was measured by densitometry analysis using image J software. cMYC/GAPDH ratio of sham treated cells was considered as unity and relative cMYC/GAPDH ratios were calculated (plotted on the top of the blot). The results show that treatment of Saos2 cells with 5-AzadC and 4-PBA decreased endogenous cMYC levels. **B**) Cells were treated with 5-AzadC and 4-PBA at concentrations of i) 0 µM & 0 mM; ii) 0 µM & 3 mM; iii) 1.5 µM & 1.5 mM; iv) 1.5 µM & 3 mM; v) 3 µM & 1.5 mM; vi) 3 µM & 0 mM; and vii) 3 µM & 3 mM, respectively. Green and red color staining represents live and dead cells, respectively. **C**) FACS analysis of cells treated with 5-AzadC and 4-PBA at various dose combinations led to apoptosis (% given in parenthesis). FACS analysis of cells treated with 5-AzadC and 4-PBA together with Z-VAD-fmk, which led to a significant reduction in the apoptosis of treated cells shown on the right panel. **D**) Cytotoxicty assay in 5-Aza and 4-PBA treated Saos2 cells with untreated (control) and sham treated (0/0) controls. Maximum LDH release from equal number of sham treated cells grown under identical condition is shown as positive control.

We showed previously that expression of *cMYC* is inversely correlated and functionally linked to expression of 14q32 miRNAs [7]. Specifically, reintroduction of 14q32-associated miR-544, miR-369, miR-382, and miR-134 reduced steady state levels of cMYC protein and induced apoptosis of Saos2 cells. We therefore examined if restoring expression of 14q32 miRNAs using DNA and chromatin-modifying drugs would recapitulate these results. Figure 3A shows that, indeed, treatment with 5-AzadC and 4-PBA reduced the levels of cMYC protein in Saos2 cells significantly, and to a greater extent than either drug alone. Similarly, the combination of 5-AzadC and 4-PBA induced dose-dependent apoptosis of Saos2 cells. Saos2 cells treated with vehicle alone showed minimal cell death as measured by uptake of vital dyes (∼2.8% dead cells, Figure 3B panel (i) and 3C panel (i)). Treatment with 5-AzadC at a concentration of 3 µM slightly increased the death rate (up to 9.7%, Figure 3B panel (vi) and 3C panel (i)), whereas 4-PBA used at 3 mM was more potent (up to 28.7% death, Figure 3B panel (ii) and 3C panel (i)). The combination of both drugs was additive, progressively showing a greater death rate and reaching almost 40% as the dose for each drug was increased (Figure 3B panel (vii) and 3C (i)). Death induced by treatment with 5-AzadC and 4-PBA occurred through apoptosis, as it was inhibited by the caspase inhibitor Z-VAD-fmk (Figure 3C, panel (ii)), and there was no significant release of LDH into the medium, indicating these drugs did not induce necrotic cell death at the observed doses (Figure 3D).

### Treatment with 5-AzadC and 4-PBA restore normal gene expression profiles in Saos2 cells

**Figure 4:**
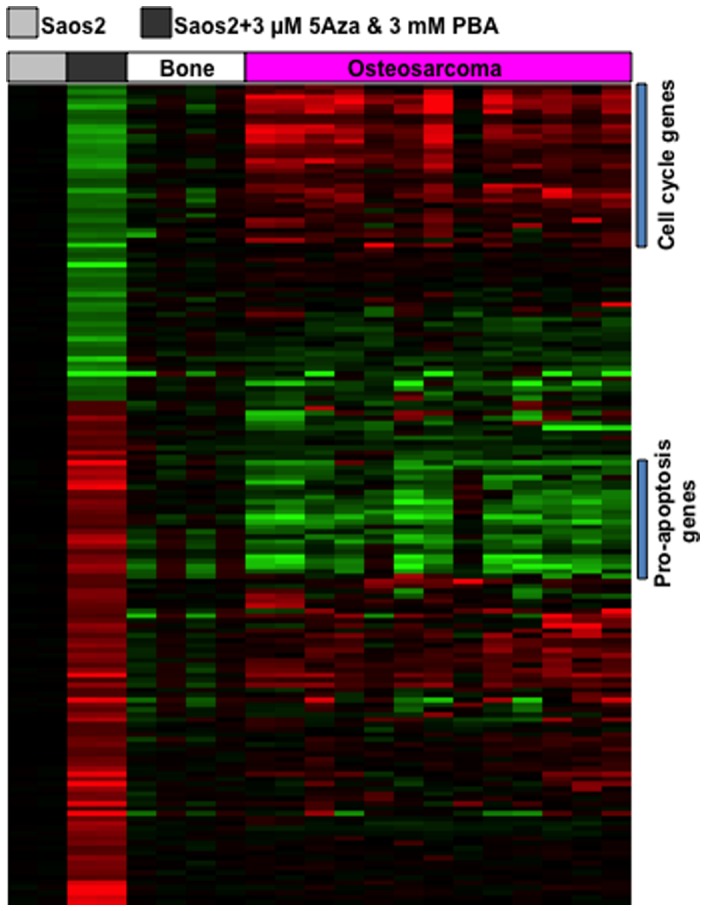
Genome-wide gene expression changes induced by treatment of 5-AzadC and 4-PBA in Saos2 cells. mRNA expression patterns in Saos2 cells treated with 3.0 µM 5-AzadC and 3.0 mM 4-PBA relative to sham treated Saos2 cells. mRNA expression profiles in normal bone (n = 4) and OS patient samples (n = 13) were used for comparison and shown relative to normal bone. The heatmap with gene names are detailed in Figure S2.

Downregulation of 14q32 miRNAs is associated with peculiar profiles of gene expression where cell cycle associated genes are overexpressed and pro-apoptotic genes are downregulated [18]. This association was clinically significant, providing accurate predictions for survival in both humans and dogs with spontaneous OS [18], [19]. Thus, we examined if treatment with 5-AzadC and 4-PBA would reverse this gene expression signature in Saos2 cells to assess the potential to utilize these compounds in the therapeutic setting. Saos2 cells were treated with vehicle or with the same combinations of 5-AzadC and 4-PBA as reported earlier [25]; and genome-wide gene expression was evaluated. To identify gene expression changes modulated by this drug combination, we compared gene expression profiles of 4 normal bone tissues and 13 OS patient samples with the gene expression profile of drug treated and untreated Saos2 cells. The analyses showed that 165 genes were differentially expressed (>2-fold change with p values <0.001, Figure 4). In the drug treated Saos2 cells, the differential gene expression patterns included downregulation of significant numbers of genes related to cell cycle function and increased expression of pro-apoptotic genes. For instance, *AURKA*, *MCM2* and *CCNB2* which are among the prototypical genes showing overexpression in the most aggressive osteosarcomas [19] were among the genes that were significantly reduced in OS cells treated with 5-AzadC and 4-PBA (Figure S2; Table 1). Of note, we also observed that LSAMP, a tumor suppressor (downregulated in 64% OS cases; deleted at single allele in 75% OS cases) [29], was also induced in drug-treated Saos2 cells. This led us to determine how drug-responsive transcripts varied in comparison to normal bone tissues. Thirty-four genes, including CDKN1A (p21), which is a transcriptional target of TP53, clustered with LSAMP showing increased expression following drug treatment (Figure S2).

**Table 1:** List of differentially expressed genes in 5-AzadC and 4-PBA treated Saos2 cells.

Cell Function	Genes	p-value
**Cell cycle genes downregulated**	*AURKA, MCM2, MCM7, MCM10, CCNB2, CDC25B, CDC45L, CCDC25, COL1A1, TYMS, KNTC1, CENPF, MELK, NASP, UBE2C, FANCD2, CHEK1, DNMT1*	3.76E-07
**Pro-apoptotic genes upregulated**	*CDKN1A, LSAMP, IL8, TRIB3, FBXO32, EGR2, GDF15, DUSP1, VIP, NLRP3, NR4A2, CDKN1A, BEX2, HERPUD1, PTGS2, RIPK2, SRXN1, ABCB1, HEY1, SLC2A3, ACVR1, ASNS, GEM, PTHLH, RGS4, CCL2, REPS2, LRIG1, BIRC3, SLIT2, STAT1, NEK6*	1.49E-08

### The cytotoxic effect of chromatin-modifying drugs is evolutionarily conserved and more pronounced in OS cells with aggressive biological behavior

**Figure 5:**
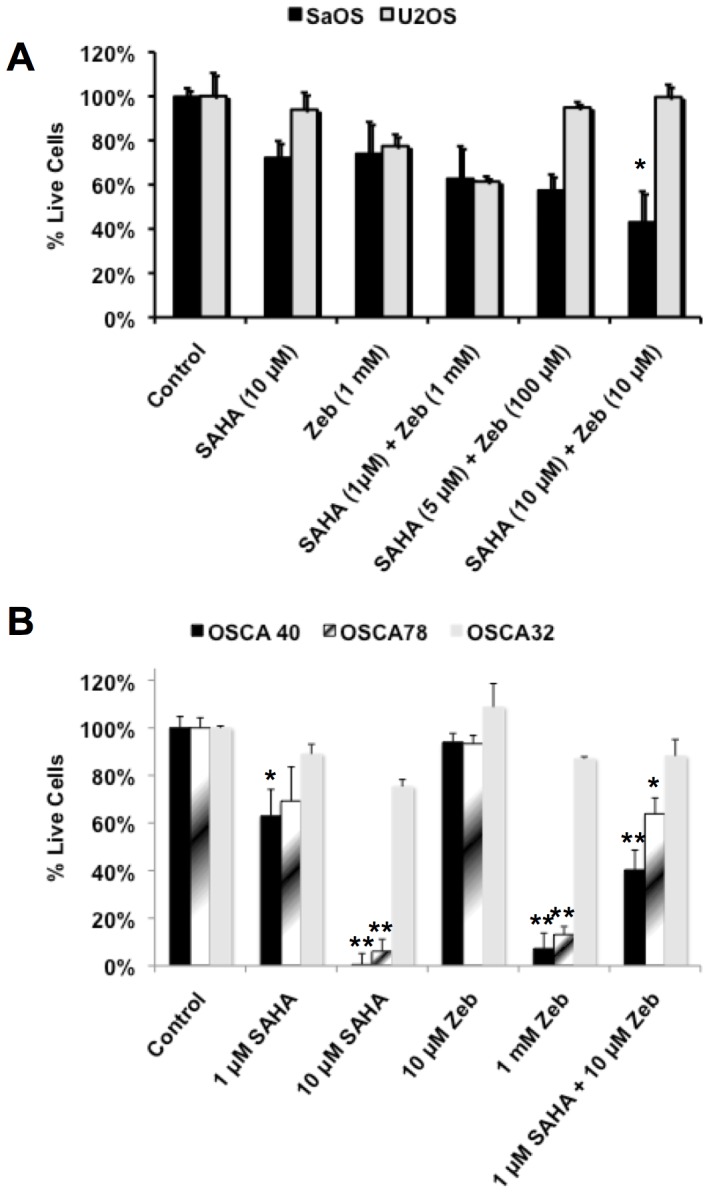
SAHA and Zeb show additive cytotoxicity in human and in canine OS cells with aggressive biological behavior. **A**) Human Saos2 and U2OS cells were cultured in the presence of SAHA and Zeb at the indicated concentrations at 48 hr. Cell viability was measured at 48 hrs using the MTS assay. Data are normalized hence100% viability reflects the number of cells present in control cultures (treated with vehicle only). **B**) Canine OSCA-40, OSCA-78, and OSCA-32 cells were cultured in the presence of SAHA and Zeb at the indicated concentrations and cell viability was measured as in panel A. Data show means ± SD for three independent experiments, performed in triplicates. Data for each condition were normally distributed with <15% intra- and inter-experimental variation from the mean. Conditions that were significantly different from control by paired Student's t-test are denoted by asterisks (* p≤0.05; ** p≤0.01).

Several chromatin-modifying drugs have been approved for clinical use by the FDA, providing an opportunity to confirm the cytotoxic effects of these compounds in OS using drugs with established profiles of efficacy and toxicity. Our comparative resources also provided an opportunity to test the hypothesis that these compounds would be most effective against tumors with highly aggressive behavior. Specifically, there was an inverse correlation between downregulation of 14q32 miRNAs and the associated gene expression profile and clinical outcome in dogs and in humans with OS [18], [19]. Using gene expression profiles as predictors for biological behavior, we expected that human Saos2 cells would show a dramatic response to chromatin modifying drugs, whereas the response in U2OS cells would be muted. Similarly, we would expect that canine OSCA-40 and OSCA-78 cells (more aggressive) would show a dramatic growth inhibition in response to chromatin modifying drugs, whereas response of OSCA-32 (less aggressive) would be muted. We tested this hypothesis using the alternate histone deacetylase inhibitor, SAHA, and the DNA methylation inhibitor Zeb, both of which are approved for clinical use in humans. In general, cell lines with more ‘aggressive’ gene expression profiles and shorter doubling times from both species were more sensitive to the effects of both compounds (Figure 5), and the response was more pronounced in canine cells. Next we adjusted drug combinations to test for additive or synergistic cytotoxicity at minimally effective concentrations for cells in each species. The results conformed to our prediction that sensitivity to chromatin modification would be directly related to specific patterns of genome-wide gene expression and downregulation of 14q32 miRNAs. Figure 5A shows that additive and significant cytotoxicity was achievable by subtherapeutic combinations of SAHA and Zeb in Saos2 cells, but not U2OS cells. Figure 5B similarly shows that additive and significant cytotoxicity was achievable by subtherapeutic combinations of SAHA and Zeb in OSCA-40 and OSCA-78 cells, to a lesser extent in OSCA-32 cells.

## Discussion

Human 14q32 genomic locus is rich in miRNAs [11]; and the majority of miRNAs at this locus are significantly downregulated in OS compared to normal bone tissues [7], [8]. Previous experiments using high-resolution aCGH showed no recurrent DNA copy number losses at 14q32 locus [7]. In this study, we show that epigenetic modifications can be attributed to the dysregulation of these miRNAs and their expression can be restored in OS cells by a combination of DNA demethylating and HDAC inhibitors.

Since there were no detectable DNA copy number changes at the 14q32 locus in OS, we carried out extensive DNA methylation analyses of CpG sites identified in the upstream regions of miRNA clusters at 14q32 region. We expected to see hypermethylation patterns at the promoter regions of these CpG sites; and, in contrast, methylation patterns at majority of these predicted promoter regions showed either no methylation changes or hypomethylation patterns compared to methylation patterns of age matched normal bone tissues. Earlier we have shown that the paternally and maternally expressed genes and miRNAs in this locus are all significantly downregulated [7]. Furthermore, we observed significant downregulation of miRNAs at 12qF1 (homologous to 14q32) in *Sleeping Beauty* induced spontaneous model of mouse OS [30]. These observations suggested a master control mechanism that potentially regulates the entire 14q32 miRNA region that spans about 200 kb. In support of this hypothesis, a recent study using mouse induced pluripotent stem cells showed that miRNAs at 12qF1 were significantly downregulated due to hypermethylation in the corresponding differentially methylated regions (DMRs) [31]. Further, homologous region of *14q32* miRNA clusters in mice is maternally expressed and related to the surrounding imprinted genes such as *Dlk1*, *Gtl2* and *Rtl1* which are also involved in pUPD12 and pUPD14 syndromes [32].

Although DNA methylation patterns in the promoter regions of 14q32 miRNA cluster did not provide a definitive answer for the downregulation of miRNAs at this locus, our ChIP assays using Saos2 cells, revealed a role for histone modification(s) in the regulation of these 14q32 miRNAs. Histones control the chromatin structure and gene expression through post-translational modifications such as acetylation [33]. Hence it is possible that deregulations in both DNA methylation and histone modification can jointly regulate the 14q32 miRNAs and imprinted genes such as *DLK1* and *MEG3* at this locus.

Histone deacetylase (HDAC) inhibitors along with other drugs have shown a significant effect in targeting cancer cells [34]–[37]; and HDAC inhibitor valproic acid in combination with other drugs has been shown to be effective against advanced solid tumors in phase I trials [38]. However, there are only limited studies where epigenetic modifying drugs were tested against OS. For instance, inhibition of HDAC in OS was shown to be effective in targeting OS cells both *in vitro* and *in vivo* by increasing the sensitivity to Fas-mediated cell death [39], [40]; and HDAC inhibition through valproic acid sensitized human and canine OS to doxorubicin [41].

It is especially relevant that DNA and chromatin-modifying drugs lead to apoptosis of OS cells *in vitro* in our studies, and a recent report documented the chemosensitization of OS cells *in vitro* and in xenograft models by the HDAC inhibitor valproic acid [41]. Herein, we showed that specific doses of 5-AzadC and PBA increased the apoptosis of Saos2 cells. Furthermore, combinatorial treatment with these DNA and chromatin-modifying drugs also activated miRNAs at the 14q32 locus and significantly lowered the expression of cell cycle genes in treated Saos2 cells. Treatment of Saos2 cells with the drug combination reversed the expression of cell cycle and pro-apoptotic genes. Comparison of gene expression profiles of normal bone tissue, OS tumor tissues and drug treated OS cells along with their controls revealed that drug treatment can potentially revert Saos2 cells to mimic a normal bone tissue gene expression pattern. In a previous study, treating U2OS cells with decitabine (demethylating agent) also resulted in an increased expression of pro-apoptotic genes such as *GADD45A, HSPA9B, PAWR, PDCD5, NFKBIA* and *TNFAIP3* [42]. Although, we noticed significant expression of many pro-apoptotic genes in our study, expression of the above mentioned genes were not significantly altered. This could be attributed to cell line specificity and the selective targeting of genes by combinatorial drug treatments.

OS tumors can be distinguished based on a cell cycle signature into two groups with differential survival probabilities [19]. Expression signatures of human and canine OS samples are comparable [18], [19] and reversion in the expression of such conserved key signature genes upon drug treatment has been observed in the current study. In a recent study [41], treatment of valproic acid downregulated the expression of key genes involved in cell cycle proliferation such as Aurora A kinase, Cyclin A in canine OS cell lines which is consistent with our study. These observations suggest that the DNA and chromatin-modifying drugs used in this study work through evolutionarily conserved mechanisms and could offer effective treatment options.

It is worth noting that in OS (and perhaps in other types of tumor) an approach that combines HDAC inhibition and demethylating agents might only improve outcome in patients with more aggressive disease (worse prognosis). Since these drugs are not without side effects, this prediction should be tested clinically in OS, using a limited gene expression signature and/or 14q32 miRNA levels as surrogates for prognosis with combination therapies to inhibit or reverse the activity of HDACs and DNA methyltransferases. Existence of FDA-approved drugs in both classes makes such trials feasible. Moreover, the availability of a spontaneous canine OS model where both the molecular pathogenesis and the clinical heterogeneity of the disease are evolutionarily conserved with humans, and where OS is diagnosed with significantly greater frequency, provides a realistic avenue to test the implication that this therapy would be best incorporated into the clinic if used with predictive tests and applied only to the cases with worse prognosis. Preclinical models will allow assessment of *in vivo* effects of these drugs in combination, as well as the potential for chemosensitization, which may lead to novel treatment therapies for OS.

## Supporting Information

Figure S1 **Controls for the COmbined Bisuifite Restriction Analysis (COBRA).** The pGEMT vector (GE healthcare, USA) was used as a control DNA to monitor the activity of restriction enzymes. *Alu*I and *Mse*I were used as testers for successful bisulfite-mediated conversion. *Alu*I (AGCT) – original recognition site should be eliminated by bisulfite- mediated conversion; *Mse*I (TTAA) – new recognition sites could be generated from CCAA, CTAA, or TCAA by bisulfite treatment.
(PDF)

Figure S2 **Genome-wide gene expression changes induced by 5-AzadC and 4-PBA treatment in Saos2 cells (Saos2_4A-1 and 4A-2) compared to vehicle treated (Saos2_0A & Saos2_0B) along with OS patients (FT-12, FT-13, FT-14, FT-15, FT-16, FT-17, FT-18, FT-19, FT-2, FT-3, FT-5 and FT-7) and normal bone (FT-406-1, FT-406-2, FT-407-1 and FT-407-2) samples are shown.**
(PDF)

Table S1 List of oligos used for combined bisulfite restriction analyses.
(PDF)

Table S2 List of oligos used for ChIP assay.
(PDF)

Table S3 List of oligos used for qRT-PCR experiments.
(PDF)
