# Supplementary figures and images for: Combinatorial Treatment of DNA and Chromatin-Modifying Drugs Cause Cell Death in Human and Canine Osteosarcoma Cell Lines
Source: PLoS One. 2012 Sep 5;7(9):e43720. doi: 10.1371/journal.pone.0043720 (PMC3434163; doi:10.1371/journal.pone.0043720)

**Supplementary  
Figure S1**

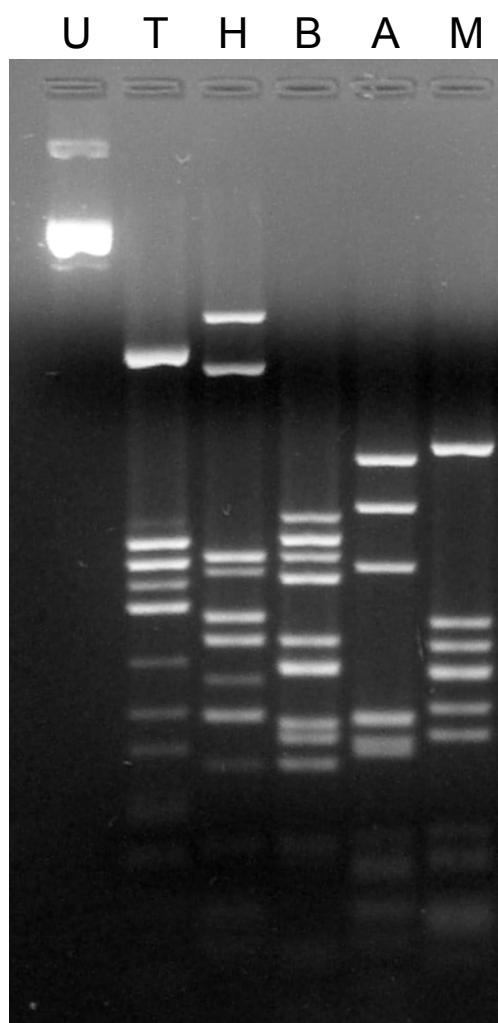

Supplement: Figure S1 — Controls for the COmbined Bisuifite Restriction Analysis (COBRA). The pGEMT vector (GE healthcare, USA) was used as a control DNA to monitor the activity of restriction enzymes. AluI and MseI were used as testers for successful bisulfite-mediated conversion. AluI (AGCT) – original recognition site should be eliminated by bisulfite- mediated conversion; MseI (TTAA) – new recognition sites could be generated from CCAA, CTAA, or TCAA by bisulfite treatment. (PDF) [file pone.0043720.s001.pdf]

# Supplementary Figure S2

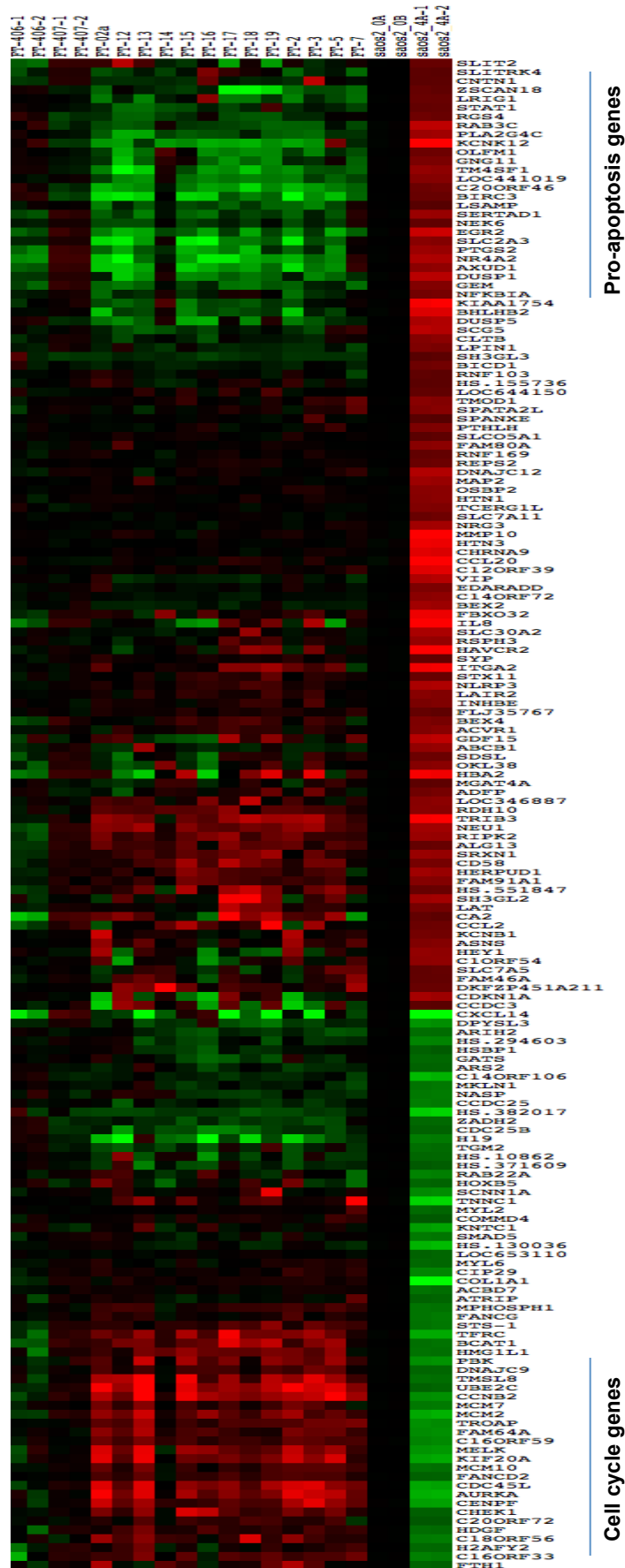

Supplement: Figure S2 — Genome-wide gene expression changes induced by 5-AzadC and 4-PBA treatment in Saos2 cells (Saos2_4A-1 and 4A-2) compared to vehicle treated (Saos2_0A & Saos2_0B) along with OS patients (FT-12, FT-13, FT-14, FT-15, FT-16, FT-17, FT-18, FT-19, FT-2, FT-3, FT-5 and FT-7) and normal bone (FT-406-1, FT-406-2, FT-407-1 and FT-407-2) samples are shown. (PDF) [file pone.0043720.s002.pdf]
