# Supplementary material for: Combinatorial Treatment of DNA and Chromatin-Modifying Drugs Cause Cell Death in Human and Canine Osteosarcoma Cell Lines
Source: PLoS One. 2012 Sep 5;7(9):e43720. doi: 10.1371/journal.pone.0043720 (PMC3434163; doi:10.1371/journal.pone.0043720)
Supplement: Table S1 — List of oligos used for combined bisulfite restriction analyses. (PDF) [file pone.0043720.s003.pdf]

Supplementary

Table S1: List of oligos used for Combined bisulfite restriction analyses

431 forward	5’-GTTGTTGTGTGGTTGGGTGGGGT-3’
431 reverse	5’-CCCAAAAACTAACTAAACTATTCC-3’
127 forward	5’-GGTTTTTGTTTAGGGAGTAGTAG-3’
127 reverse	5’-CAAAACTACATACAAATAATTCC-3’
432 forward	5’-TTTATTTGGTTAGTTTAAGATGG-3’
432 reverse	5’-AAAATCAATACAAACCAACTACC-3’
411 forward	5’-GTATTTTTGTGTGGTATTTGGAG-3’
411 reverse	5’-AACTAAAAAAAAACTCATATCCAC-3’
