# Supplementary material for: Combinatorial Treatment of DNA and Chromatin-Modifying Drugs Cause Cell Death in Human and Canine Osteosarcoma Cell Lines
Source: PLoS One. 2012 Sep 5;7(9):e43720. doi: 10.1371/journal.pone.0043720 (PMC3434163; doi:10.1371/journal.pone.0043720)
Supplement: Table S2 — List of oligos used for ChIP assay. (PDF) [file pone.0043720.s004.pdf]

Supplementary
Table S2: List of oligos used for ChIP assay

	**Nucleotide sequence**
miR-544chip1-F	5’-TTGGATTATTCTTGGTGGACGTGGCATG-3’
miR-544chip1-R	5’-CTTAGGAGCATTTTTAAAGTTTGAGAAACACTAC-3’
miR-369chip1-F	5’-TCTTGGAGGCTGGGGCACC-3’
miR-369chip1-R	5’-GTACCAGGAAAGGCGCTGAGCT-3’
miR-382chip1-F	5’-AGGGAAGGGGGACTGTGCC-3’
miR-382chip1-R	5’-CCACAGAAAAGCAGAGACAAGACAGAC-3’
miR-134chip1-F	5’-CAATGCGCTGACCTAGCTGTAAGTC-3’
miR-134chip1-R	5’-GACGGTGCTGACACCAACATCTCTTC-3’
miR-654chip1-F	5’-GTTTGATGGATTGTACTTAGGTTCGTG-3’
miR-654chip1-R	5’-GATTAACCAGGAATCACGTAA ACATAGAAGGAA-3’
miR-431chip1-F	5’-GCCTGTAGATCAGGGTCAGGA AC-3’
miR-431chip1-R	5’-CCAGCCCAGGAGCTGGC-3’
miR-127chip1-F	5’-CCCACGCAGCTCCAGTTTTG-3’
miR-127chip1-R	5’-CATCCGCTGGCTCCGAGT-3’
miR-432chip1-F	5’-GACCTCACCATGGCCATAAGTTCTG-3’
miR-432chip1-R	5’-TCTGAAGACTCATTTGAGACGATGATGG-3’
miR-411chip1-F	5’-AGGAGTTGCCAGTTTTGGGG-3’
miR-411chip1-R	5’-TTCCGGGAAGAATCA AGG TCTCC-3’
GAPDHchip1-F	5’-TACTAGCGGTTTTACGGGCG-3’
GAPDHchip1-R	5’-TCGAACAGGAGGAGCAGAGAGCGA-3’
CHIPnegative-F	5’-ATGGTTGCCACTGGGGATCT-3’
CHIPnegative-R	5’-TGCCAAAGCCTAGGGGAAGA-3’
