# Supplementary material for: Combinatorial Treatment of DNA and Chromatin-Modifying Drugs Cause Cell Death in Human and Canine Osteosarcoma Cell Lines
Source: PLoS One. 2012 Sep 5;7(9):e43720. doi: 10.1371/journal.pone.0043720 (PMC3434163; doi:10.1371/journal.pone.0043720)
Supplement: Table S3 — List of oligos used for qRT-PCR experiments. (PDF) [file pone.0043720.s005.pdf]

Supplementary Table S3: List of oligos used for qRT-PCR experiments

Name and miRbase number of the microRNA	Oligo sequence
hsa-miR-410 MIMAT0002171	5’ -GGGCAATATAACACAGATGGCCTGT-3’
hsa-miR-369-3p MIMAT0000721	5’ -GGGGGGAATAATACATGGTTGATCTTT-3’
hsa-miR-382 MIMAT0000737	5’ -GGGGAAGTTGTTCGTGGTGGATTCG-3’
hsa-miR-544 MIMAT0003164	5’ -GGGGGATTCTGCATTTTTAGCAAGTTC-3’
hsa-miR-127-5p MIMAT0004604	5’ -GGCTGAAGCTCAGAGGGCTCTGAT-3’
hsa-miR-154 MIMAT0000452	5’ -GGAGGTTATCCGTGTTGCCTTCG-3’
hsa-miR-134 MIMAT0000447	5’ -TGTGACTGGTTGACCAGAGGGG-3’
